# Functional validation of transposable element–derived *cis*-regulatory elements in Atlantic salmon

**DOI:** 10.1093/g3journal/jkad034

**Published:** 2023-02-08

**Authors:** Hanna M Sahlström, Alex K Datsomor, Øystein Monsen, Torgeir R Hvidsten, Simen Rød Sandve

**Affiliations:** Department of Animal and Aquacultural Sciences, Faculty of Bioscience, Norwegian University of Life Sciences, 1430 Aas, Norway; Department of Animal and Aquacultural Sciences, Faculty of Bioscience, Norwegian University of Life Sciences, 1430 Aas, Norway; Department of Animal and Aquacultural Sciences, Faculty of Bioscience, Norwegian University of Life Sciences, 1430 Aas, Norway; Faculty of Chemistry, Biotechnology and Food Science, Norwegian University of Life Sciences, 1430 Aas, Norway; Department of Animal and Aquacultural Sciences, Faculty of Bioscience, Norwegian University of Life Sciences, 1430 Aas, Norway

**Keywords:** transposable elements, whole-genome duplication, gene expression, evolution, Atlantic salmon

## Abstract

Transposable elements (TEs) are hypothesized to play important roles in shaping genome evolution following whole-genome duplications (WGDs), including rewiring of gene regulation. In a recent analysis, duplicate gene copies that had evolved higher expression in liver following the salmonid WGD ∼100 million years ago were associated with higher numbers of predicted TE-derived *cis*-regulatory elements (TE-CREs). Yet, the ability of these TE-CREs to recruit transcription factors (TFs) in vivo and impact gene expression remains unknown. Here, we evaluated the gene-regulatory functions of 11 TEs using luciferase promoter reporter assays in Atlantic salmon (*Salmo salar*) primary liver cells. Canonical Tc1-*Mariner* elements from intronic regions showed no or small repressive effects on transcription. However, other TE-CREs upstream of transcriptional start sites increased expression significantly. Our results question the hypothesis that TEs in the Tc1-*Mariner* superfamily, which were extremely active following WGD in salmonids, had a major impact on regulatory rewiring of gene duplicates, but highlights the potential of other TEs in post-WGD rewiring of gene regulation in the Atlantic salmon genome.

## Introduction

Whole-genome duplication (WGD) is believed to result in functionally redundant genes, which can evade selective constraints and thereby evolve novel functions and regulation (Ohno 1970). In vertebrates, a series of recent studies across 4 ancient WGDs have revealed that regulatory divergence is extensive and mostly asymmetric following WGD (Lien *et al.* 2016; Marlétaz *et al.* 2018; Sandve *et al.* 2018), with 1 gene copy retaining an ancestral-like regulation while the other copy evolves novel-regulatory phenotypes. Yet, the underlying mechanisms driving evolution of novel-regulatory phenotypes are mostly unknown.

Salmonid fish underwent a whole-genome duplication ∼100 million years ago, and presently about 50% of the retained gene duplicates have diverged regulation (Lien *et al.* 2016). In a recent study (Gillard *et al.* 2021), we used a comparative phylogenetic approach to explore the impact of WGD on adaptive evolution of gene expression in the liver. Using the salmonids as a study system, we found that WGD boosted gene expression evolution, with the majority of duplicate pairs evolving lower expression in one of the duplicates across many tissues. These genes also showed significant enrichment of transposable element (TE) insertions in the promoter region. Only a very small fraction of duplicated genes (30 pairs) evolved liver-specific increase in expression in 1 copy, as expected under a scenario of adaptive evolution of novel gene functions following WGD. Interestingly, these candidates showed strong signatures of gains in TF-binding sites (TFBSs) for liver-specific TFs. Furthermore, TFBSs predicted to be bound by liver-specific TFs overlapped TEs more often in the evolved copy. These findings hint to a role of TEs in gene-regulatory rewiring following WGD.

TE activity can have a large impact on evolution of TFBSs and *cis*-regulatory landscapes (Chuong *et al.* 2017; Sundaram and Wysocka 2020) and are sometimes co-opted by their hosts (Chuong *et al.* 2017). In the short term, functional and active TEs can directly donate sequence motifs embedded in the TE that functions as TFBSs in the host genome. At a longer time scale, TE insertions can accumulate secondary mutations that can result in de novo evolution of *cis*-regulatory elements (CREs). In Atlantic salmon, TEs make up a large fraction of the genome (about 51%), with the largest superfamily of Tc1-*Mariner* DNA transposons experiencing an expansion coinciding with the WGD (Lien *et al.* 2016). This has further fueled the hypothesis that TEs have played a key role in sequence and regulatory divergence. Yet the potential of specific TEs as CREs in salmonids is still unknown.

In this study, we aim to test the hypothesis that TEs play a role in tissue-specific expression divergence among duplicate gene copies from the salmonid WGD. We do this using luciferase promoter reporter assays to test the *cis*-regulatory activity of TEs predicted to be bound by liver-biased TFs identified in Gillard *et al.* (2021).

## Materials and methods

### TE selection for reporter-assay characterization

Gillard *et al.* (2021) identified 30 Atlantic salmon gene duplicate pairs where 1 copy had evolved a liver-specific increase in expression following WGD. Among those genes, several of the promoter regions were predicted to be bound by liver-active TFs at TFBSs within sequences annotated as TEs. Using this data set as a starting point, we first identified Tc1-*Mariner* insertions that were candidates for being CREs and drive gene transcription in the liver, including. Of these, 5 Tc1-*Mariner* elements were selected for reporter assays; SsalEle0180, SsalEle0256, SsalEle0351, SsalEle0401, and SsalEle0709. Following PCR amplification of the TE insertions, only 4 were used in the experiments due to PCR failure of SsalEle0256. These TE insertions were only present in the promoter of the gene duplicate copy with diverged expression.

When selecting TE-derived CREs (TE-CREs) from TEs other than Tc1-*Mariners* for reporter assays, we used the same list of putative TE-CREs as used to select the Tc1-*Mariner* elements. We required TE sequences to be >100 bp and only present in the promoter region (−500/+200 bp from transcription start site) of the gene copy with high liver expression. This resulted in 7 TE-CREs with a putative role in evolution of increased liver expression. One of these TEs contained a nested insertion of another TE fragment, both part of the OM_rnd-6 family-242, and these were tested together. TE-CRE filtering steps were carried out in R and the code can be found at https://gitlab.com/hansahls/te_reg_repository.

### TE characterization

Manual curation of the selected TE insertions’ sequences was carried out using a pipeline described in Suh *et al.* (2018): Blastn was first used to identify similar sequences in the genome. We selected the genomic regions of the 20 best hits and extracted these regions (±2 kb) as fasta sequences fasta format using the getfasta function in bedtools v2.18 (Quinlan and Hall 2010), aligned sequences using MAFFT (Katoh and Standley 2013), and visually inspected the alignments. TEs were characterized according to terminal repeats, ORF compositions, known characteristic motifs and Repbase similarity searches (Bao *et al.* 2015).

To estimate a relative measure of the transposition activity for the TE subfamiles, we used RepeatMasker (v. 4.1.0; Smit *et al.* 2015) to compute the sequence similarity between TE insertions and their consensus TE sequence. We estimated the relative age (per cent similarity at the base pair level) of the TE copies used in reporter assays by blasting the fasta for each TE insertion sequence to its consensus TE sequence.

### Phylogenetic analysis of Tc1-*Mariner* transposable elements

To get an overview of the relatedness and subclassification, we conducted a phylogenetic analysis of the Tc1-*Mariner* TEs (SsalEle0180, SsalEle0256, SsalEle0351, SsalEle0401, and SsalEle0709). DNA sequences of the TEs were extracted from the salmon genome sequence (NCBI: ICSASG v2) in the fasta format using the getfasta function in bedtools v2.18 (Quinlan and Hall 2010). The TE sequences were then combined with all Tc1-*Mariner* consensus sequences from (Lien *et al.* 2016). TEs shorter than 100 bp were disregarded, and the sequences were then aligned using MAFFT v7.475 (Katoh and Standley 2013) with the parameters –adjustdirection and –auto. A phylogenetic tree was then estimated from the multiple sequence alignment using FastTree v2.1.11 (Price *et al.* 2010). This tree was then visualized using the ggtree package (v2.2.4; Yu *et al.* 2016) in R. The code for this phylogenetic analysis can be found at https://gitlab.com/hansahls/te_reg_repository/-/blob/main/Phylogenetic_Analysis.Rmd.

### Preparation of luciferase reporter constructs

To validate the transcriptional regulatory roles of the putative TE-CREs found to be associated with liver-specific duplicate gene expression, we performed luciferase reporter assays. The reporter vectors were constructed in 2 different ways, using PCR amplicons or oligo-synthesis. For 5 Tc1-*Mariner* elements, we PCR-amplified whole TE elements or subregions of the TEs from Atlantic salmon genomic DNA and cloned these into vectors for reporter assays. PCRs were done using Platinum SuperFi PCR Master Mix (Invitrogen) using primers that also bear 15 bp tail sequences homologous to *Sac*I and *Xho*I restriction enzyme cloning sites within the pGL3-Promoter firefly luciferase vector (Promega, GenBank Accession number U47298). All PCR primers are listed in Supplementary File 1. PCR-amplified elements were gel-purified using the QIAquick Gel Extraction Kit (Qiagen #28706) and cloned into *Sac*I- and *Xho*I-digested pGL3-Promoter vector using the InfusionHD Cloning kit (Takara Bio #639650). Elements were cloned upstream of the sv40 promoter within the pGL3-Promoter vector. Vector design and map files can be found in Supplementary Files 2–6. Recombinant reporter constructs were isolated using the ZymoPURE Plasmid Miniprep Kit (Zymo Research, #D4210), following the manufacturer's protocol with the following minor modifications. 50 ml tubes were centrifuged at 4000 rpm (1700 x g) for 5 min, and the supernatant was discarded. 500 μl of ZymoPure P1 (Red) was used for pellet resuspension, and then transferred to a 3.0 ml Lo-bind tube. 500 μl of ZymoPURE P2 and ZymoPURE P3 was used instead of 250 μl. 600 μl of lysate was transferred to 2 tubes (total volume 1200 μl). Final elution centrifugation was done at 11,000*×g* for 3 min instead of 1 min. After elution the DNA concentrations were measured using a Nanodrop 8000 spectrometer.

Another set of 6 potential TE-CREs were synthesized and cloned within *Sac*I- and *Xho*I-linearized pGL3-Promoter vector (outsourced to Genscript). All reporter constructs were confirmed by Sanger sequencing (LightRun Tube Sequencing Service, Eurofins).

### Transfection of salmon primary liver cells and Dual-Glo luciferase assay

We reasoned that Atlantic salmon primary hepatocytes are ideal for validating the roles of the TEs in liver-specific regulation of duplicated genes owing to the lack of salmon liver continuous cell lines, and to the fact that they are of liver origin. Primary hepatocytes were isolated using a protocol optimized by Datsomor *et al.* (2022). Cells were isolated from Atlantic salmon with an average weight and length of 30.3 g and 310.9 cm, respectively. Approximately 1.0–1.5 × 10^5^ primary hepatocytes were co-transfected per well in 24-well plates with 1.7 μg of each Tc1-*Mariner* reporter construct together with 0.3 μg of pGL4.75[*hRluc*/CMV] (Promega) which encodes Renilla luciferase whose activity is used as an internal standard for normalizing variations in cell number and transfection efficiency. For SINE and LINE reporter constructs, 1.5 μg of each reporter construct was co-transfected with 0.5 μg of pGL4.75[*hRluc*/CMV]. Transfection was performed using the Neon transfection system with an electroporation program optimized for primary hepatic cells (Datsomor *et al.* 2022): 1400 voltage, 20 ms pulse width and 2 pulses. The primary hepatocytes were cultured at 15°C under atmospheric conditions in L15 GlutaMAX medium (ThermoFisher) supplemented with 5% fetal bovine serum (without antibiotics). Medium was replaced with fresh L15 GlutaMAX supplemented with 5% fetal bovine serum and 1× penicillin-streptomycin 24 h post-transfection and the cells cultured for additional 24 h. To assess firefly luciferase activities per well, medium on cells was replaced with 100 µl each of Dulbecco's modified Eagle's medium (Sigma) and Dual-Glo luciferase reagent (Promega) and incubated for 30–45 min. Luminescence was read on Synergy H1 Hybrid multi-mode microplate reader (BioTek). Luminescence from Renilla luciferase activities was measured 10 min after adding 100 µl of Dual-Glo Stop & Glo reagent. Firefly luminescence was normalized to Renilla luciferase luminescence and presented as means of triplicates, unless stated otherwise.

### Statistical analyses of differences in luciferase expression

Initial processing of the luciferase assay results was done according to the manufacturer's instructions (Dual-GloⓇ Luciferase Assay System, Promega, #E2920). In brief, normalization of the data was carried out by calculating the fold change between firefly RLU and renilla-RLU for each well, and then calculating the mean of all replicates. This normalization step mitigated unwanted effects from differences in transfection efficiency and cell survival variability between wells. The signals from the untransfected wells acted as a control for well contamination and other experimental issues.

A statistical test for differences in LUC expression was performed for all experiments. For the Tc1-*Mariner* experiments, LUC expression for each construct was measured in 3 replicates. Since these triplicates are from the same transfection procedure with identical conditions the errors should only be small, related to instrumental variation and normally distributed. We therefore used an ANOVA and a post hoc Tukey test. For the experiments with non-Tc1-*Mariner* TEs we replicated each luciferase experiment 3 times, with 3 biological replicates per experiment. We observed clear between-experiment variation in LUC expression, and to account for this we used a linear regression model with each experiment as a cofactor to test whether the TEs have a significant effect on luciferase expression. Scripts to reproduce statistical tests and visualizations can be found at https://gitlab.com/hansahls/master_thesis_hanna_sahlstrom/-/tree/master/R_code.

## Results

### Tc1-*Mariners* did not induce transcription

**Fig. 1.**
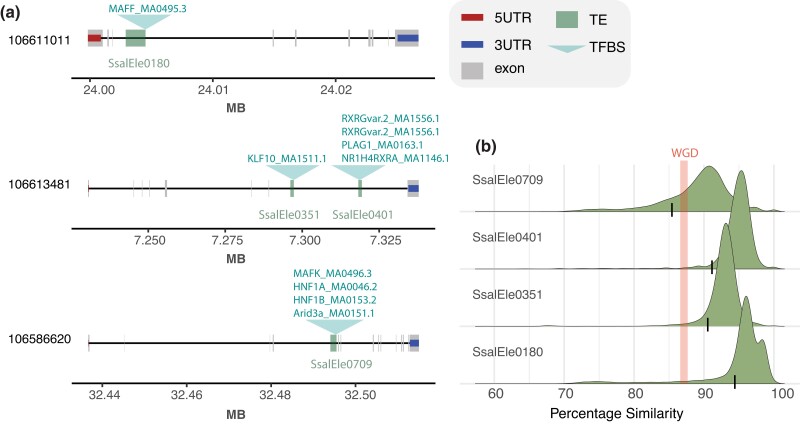
Overview of functionally validated Tc1-*Mariner* elements. a) Overview of transposable element insertion relative to gene features and predicted TFBS bound by liver-expressed TFs. Jaspar TF motif names are indicated above each TE insertion. b) Within TE-family similarity distribution reflecting age of transposition activity. TE insertions in (a) marked with a thin horizontal line. Thick line marks expected genomic similarity for duplicated genomic regions after WGD.

Gillard *et al.* (2021) found that the gene copies that had evolved increased expression levels in the liver were enriched for Tc1-*Mariner* insertions, which could act as binding sites for liver-active TFs, nearby or within the gene. To functionally evaluate whether these Tc1-*Mariner* insertions could be responsible for evolution of increased liver transcription, we cloned 4 genomic copies into LUC reporter-assay vectors (Fig. 1a). The 4 elements had the highest sequence similarity to Tc1-2, *Mariner*-22, and *Mariner*-10 families based on blast searches to RepBase and verified through visual inspections of multiple sequence alignments (Supplementary Files 8–11).

To assess the relative transposition age of TE sequences, we analyzed the distribution of TE consensus sequence similarity (Fig. 1b). The results showed that TE copy insertion similarity peaks at >90% suggesting that these TEs were active after the WGD in salmonids (average similarity between duplicated genomic regions are 87% according to Lien *et al.* 2016). However, one of the cloned element copies (SsaEle0709) had a slightly lower similarity to the consensus sequence compared with what we expected for transposition events happening post-WGD (Fig. 1b).

**Fig. 2.**
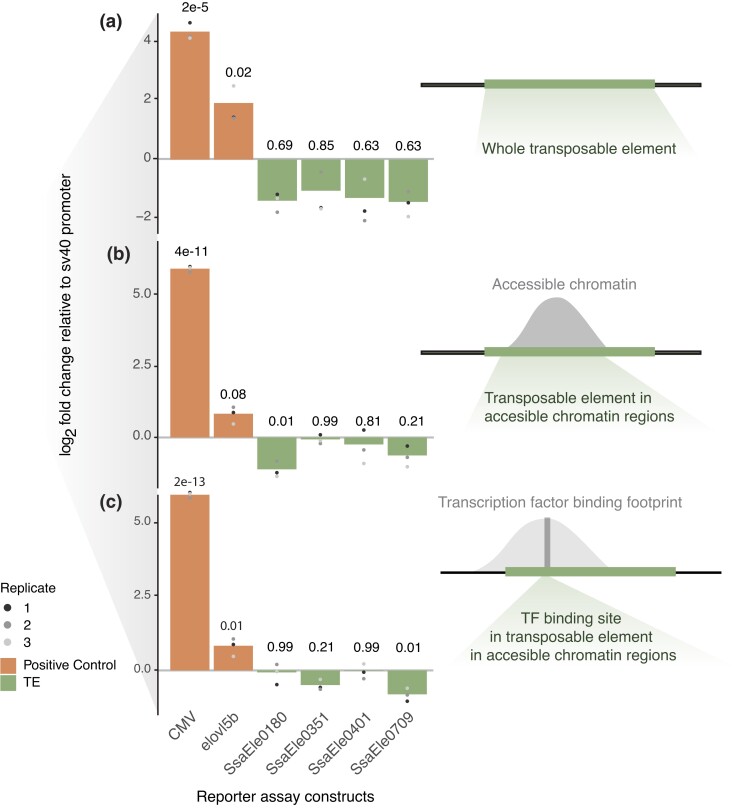
Dual luciferase reporter assays using Tc1-*Mariner* TE regions. Barplots showing log_2_ transformed mean fold change relative to sv40 empty vector across 3 experiments (a–c) with 3 replicates per experiment. a) Reporter assays testing whole TEs. b) Reporter assay testing accessible chromatin regions overlapping the TEs. c) Reporter assay testing regions of the TEs in open chromatin and predicted to be bound by liver-specific TFs. Positive controls are the CMV enhancer and the ATAC-peak closest (upstream) to the transcription start site (TSS) of the liver-expressed gene *elovl5b* (NC_059469.1; ssa28: 27245480..27256837, Assembly ICSASG_v2, Gene ID: 100192340) from Atlantic salmon.

As a first experiment to evaluate the regulatory activity of the Tc1-*Mariner* elements, we performed a LUC-reporter assay using the entire TE insertions (Fig. 2a) as CREs. Whole TE sequences did not increase expression of the LUC reporter relative to the SV40 control (Fig. 2a). Instead, we observed a non-significant reduction in luciferase signals.

In their native states in salmon liver cells, only smaller regions of these Tc1-*Mariner* elements are in accessible chromatin regions and hence can act as potential-binding sites for TFs. It is thus possible that by using whole TEs in the reporter constructs, we introduce binding of transcriptional suppressors that can cloak effects of positive regulators. To explore this hypothesis, we conducted 2 additional experiments using a nested design to exclude regions of the Tc1-*Mariner* elements likely not accessible to TFs in the native liver genome. We first tested regions of the TEs only within accessible chromatin peaks (i.e. ATAC-seq peaks), and finally, smaller regions overlapping these ATAC-seq peaks predicted to contain TFBSs bound by liver-expressed TFs (Fig. 1b and c and Supplementary File 7). Like the whole TE experiment, subregions of the Tc1-*Mariner* elements did not induce transcription compared with our SV40 negative control (Fig. 2b and c), and in 2 of the experiments, a significant repressive effect on luciferase signals were observed. In conclusion, we find no evidence supporting that the Tc1-*Mariner* elements tested here were involved in evolution of increased gene expression levels in the liver following the salmonid whole-genome duplication.

### Non-*Mariner* elements induced transcription

**Fig. 3.**
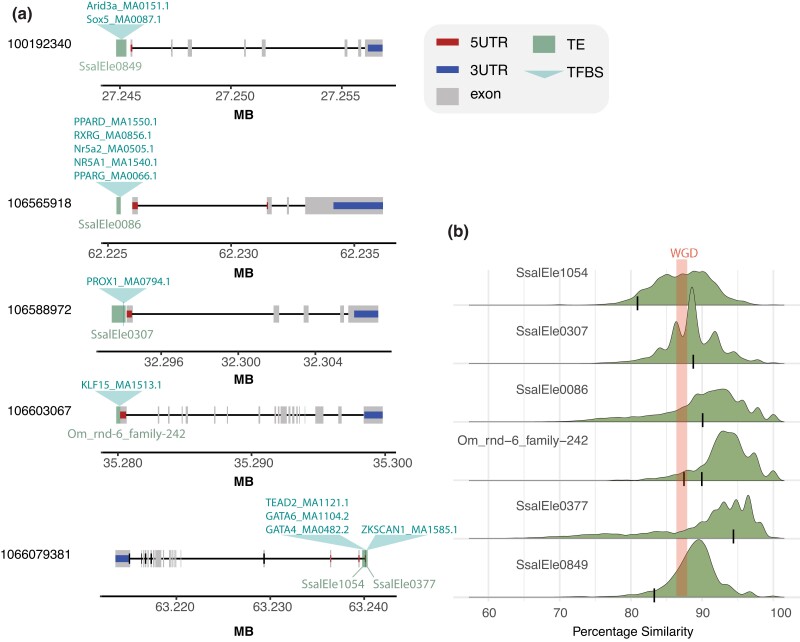
Overview of functionally validated non-*Mariner* elements. a) Overview of transposable element insertion relative to gene features and predicted TFBS bound by liver-expressed TFs. Jaspar TF motif names are indicated above each TE insertion. b) TE copy similarity distribution for each TE consensus sequence reflecting age of transposition activity. TE insertions in Fig. 3a marked with a thin line. Thick line marks expected genomic similarity for duplicated genomic regions after WGD.

**Fig. 4.**
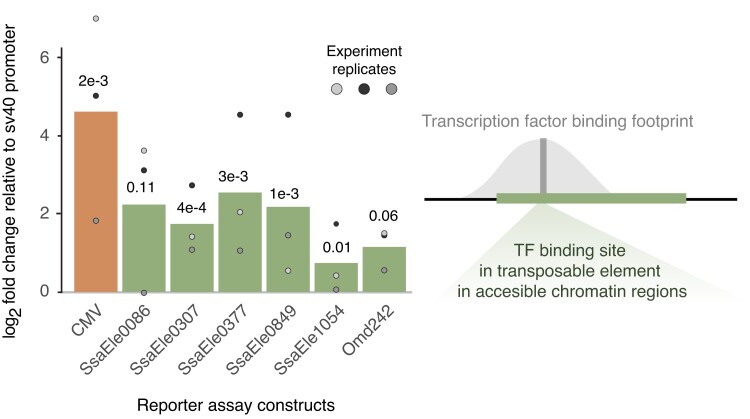
Dual luciferase assays on non-*Mariner* TE-CREs. Barplots showing log_2_ transformed mean fold change relative to sv40 empty vector across 3 replicate experiments. Mean values of 3 biological replicates within each replicate experiment are indicated with points.

Since the Tc1-*Mariner* elements associated with gene copies with increased expression levels did not induce transcription in reporter assays (Fig. 2), we decided to test other putative TE-derived CREs associated with the same genes (i.e. not from Tc1-*Mariners*). Six TE-derived sequences situated 500 bp upstream to 200 bp downstream of the TSS and carrying TFBS predicted to be occupied by liver-expressed TF were synthesized, cloned into LUC vectors, and tested for regulatory activity (Fig. 3a). The Atlantic salmon TE annotation (Lien *et al.* 2016) classified these TE-CREs as 1 LINE1 superfamily element (SsalEle0377), 1 unknown DNA transposon (SsalEle0849), and 4 TE-like sequences of unknown origin. Visual inspection of alignments (Supplementary Data Files 12–16) and blast searches to RepBase confirmed the annotation of the LINE1 superfamily element, the DTX-element annotation was considered low confidence, and the unknown TE elements were confirmed to be too degenerated to be assigned to a superfamily. The results from the LUC-reporter assays showed a clear effect of experiment with the third experiment generally showing lower LUC-expression values. Using “experiment” as a covariate in our linear model, we found significant positive effects on transcription for all constructs (*P* < 0.0013) with mean fold change ranging between 2.78 and 35.37 (Fig. 4). The SsaEle1054 had the smallest effect, while the LINE1-like SsalEle0377 had the largest effect on transcription. It is worth noting that we observed large variation in normalized luciferase signals between replicate experiments (Fig. 4); however, the rank order of effect sizes across experiments were relatively constant.

## Discussion

TEs are known to be important players in the evolution of gene regulation (Kunarso *et al.* 2010; Jacques *et al.* 2013; Sundaram *et al.* 2014; Chuong *et al.* 2017). In a recent paper, we found that gene duplicates, where 1 copy evolved higher expression in the liver after a salmonid whole-genome duplication, were enriched for insertions of Tc1-*Mariner* elements (Gillard *et al.* 2021). Although Tc1-*Mariner* elements have been shown to function as promoters and can activate transcription (Palazzo *et al.* 2019), promoter reporter experiments in this study indicated no effect or very slight repressive effects on transcription for the 4 Tc1-*Mariner* superfamily elements tested (Fig. 2a–c). This is similar to what was found in a reporter-assay experiment of primate TE-CREs in liver (Trizzino *et al.* 2017), where only 16% (mostly LTRs and DNA transposons) induced transcription. The Tc1-*Mariners* elements tested here likely did not play an important role in evolution of liver-specific divergence of gene duplicate expression as defined in Gillard *et al.* (2021). It is worth noting, however, that the Tc1-*Mariner* elements tested in this study were derived from introns (Fig. 1), and if TF-independent mechanisms such as intron-mediated enhancement (Rose *et al.* 2008; Shaul 2017) play a role in the gene duplicate divergence, our promoter–reporter assays would not be able to detect this.

The gene copies with increased liver expression also had other TEs with TFBSs predicted to bind liver-biased TFs in their promoters. Contrary to the Tc1-*Mariner* elements, these non-*Mariner* TE-CREs increased luciferase signals in our reporter assays (Fig. 4). The TE-CRE with highest transcriptional induction, SsalEle0377, is a LINE1 element, which are known to function as CREs in other species (Sundaram and Wysocka 2020). This TE-CRE only carried 1 TFBS predicted to be bound by a TF, a ZKSCAN1. Although we could not find literature supporting liver-specific function for this TFBS, the TF is highly expressed in the liver (according to GTEx) and associated with various roles in tumor development (Li *et al.* 2014; Huang *et al.* 2019). The other TE-CREs with inductive effect on transcription all contained several TFBS motifs with known roles in liver cell gene regulation and function (Fig. 4). The PPAR, Nr5a, and RXRa motifs in the SSalEle0086 element are known to play various roles in liver energy metabolism and induce transcription (Joseph *et al.* 2002; Lee *et al.* 2006; Ruggiero *et al.* 2015), while the GATA4, GATA6, and PROX1 motifs (in SsalEle1054 and SsalEle0307) are involved in liver cell fate specification and differentiation (Burke and Oliver 2002; Holtzinger and Evans 2005; Zheng *et al.* 2013).

### Conclusion

TEs are suspected of playing an important role in rewiring gene regulation in the Atlantic salmon genome after a WGD event. Our results support that TE activity has contributed to liver-specific gene duplicate divergence, but cast doubts about the importance of Tc1-mariners in gene-regulatory rewiring following the Atlantic salmon WGD. Nevertheless, a more systematic genome-wide approach is needed to reach a general conclusion regarding all Tc1-mariner elements. Plasmid-based reporter assays as used in this study also have clear limitations as they do not assay the CREs in a chromosomal context (Inoue *et al.* 2017) and cannot evaluate TF-independent gene-regulatory mechanisms. Future studies could attempt to perform CRISPR based TE-copy specific knock-out to overcome these limitations.

## Supplementary Material

jkad034_Supplementary_Data

## Data Availability

Data and code to reproduce results, as well as Supplementary files are available at figshare (https://doi.org/10.6084/m9.figshare.22001591 and https://doi.org/10.6084/m9.figshare.22001609). Data and code to reproduce results, figures are also available at https://gitlab.com/hansahls/te_reg_repository. Supplemental material available at G3 online.
