# Supplementary material for: Functional validation of transposable element–derived cis-regulatory elements in Atlantic salmon
Source: G3 (Bethesda). 2023 Feb 8;13(4):jkad034. doi: 10.1093/g3journal/jkad034 (PMC10085797; doi:10.1093/g3journal/jkad034)
Supplement: jkad034_Supplementary_Data [file jkad034_supplementary_data.zip › Supplemental_files_legends_G3-2022-403942.docx]

**Supplementary files legends**

***Supplementary file 1.*** PCR primers

***Supplementary files 2-6.*** *Vector sequence* maps of constructs used in luciferase assay analysis

**Supplementary file 7.** LUC-assay TE-sequence information.

**Supplementary files 8-17.** Sequence alignments used to confirm TE consensus annotation
